# Real-Time Emotion Classification Using EEG Data Stream in E-Learning Contexts

**DOI:** 10.3390/s21051589

**Published:** 2021-02-25

**Authors:** Arijit Nandi, Fatos Xhafa, Laia Subirats, Santi Fort

**Affiliations:** 1 Department of Computer Science, Universitat Politècnica de Catalunya (BarcelonaTech), 08034 Barcelona, Spain; arijit.nandi@eurecat.org; 2 Eurecat, Centre Tecnològic de Catalunya, 08005 Barcelona, Spain; laia.subirats@eurecat.org (L.S.); santi.fort@eurecat.org (S.F.); 3 ADaS Lab, Universitat Oberta de Catalunya, 08018 Barcelona, Spain

**Keywords:** e-learning, emotion classification, real-time emotion classification, online training, logistic regression, stochastic gradient descent

## Abstract

In face-to-face and online learning, emotions and emotional intelligence have an influence and play an essential role. Learners’ emotions are crucial for e-learning system because they promote or restrain the learning. Many researchers have investigated the impacts of emotions in enhancing and maximizing e-learning outcomes. Several machine learning and deep learning approaches have also been proposed to achieve this goal. All such approaches are suitable for an offline mode, where the data for emotion classification are stored and can be accessed infinitely. However, these offline mode approaches are inappropriate for real-time emotion classification when the data are coming in a continuous stream and data can be seen to the model at once only. We also need real-time responses according to the emotional state. For this, we propose a real-time emotion classification system (RECS)-based Logistic Regression (LR) trained in an online fashion using the Stochastic Gradient Descent (SGD) algorithm. The proposed RECS is capable of classifying emotions in real-time by training the model in an online fashion using an EEG signal stream. To validate the performance of RECS, we have used the DEAP data set, which is the most widely used benchmark data set for emotion classification. The results show that the proposed approach can effectively classify emotions in real-time from the EEG data stream, which achieved a better accuracy and *F1-score* than other offline and online approaches. The developed real-time emotion classification system is analyzed in an e-learning context scenario.

## 1. Introduction

Emotion, human intellect and learning have made intertwined relations. Emotions affect the learner’s concentration, exert their enthusiasm for learning and influence their self-regulated learning [1]. The effects of emotion, especially positive emotions, have a deeper influence on academic excellence through self-regulation of learning and motivation [2,3]. Most of the time, it is observed in electronic learning that the same lectures or even courses end up becoming boring for students. All the efforts of the instructor and their support have become wasted. Emotion promotes the activation of the associative material in the long-term memory that facilitates retrieval. Thus, during the e-learning process, it is important that the emotions of a learner are positive, which impacts learners to become more attentive and empowering to learning. As a consequence, positive emotions will enhance learner’s abilities to discover more and perform excellently in assessments, gather thorough knowledge and outshine in their career path. This convoluted relation between emotion and learning has attracted many researchers towards the study of emotion recognition in e-learning.

Conventional classroom learning has radically revolutionized into e-Learning with the aid of advanced technologies such as Massive Online Open Courses (MOOC) and Learning Management Systems (LMS). Such environments aid in the effective transfer of information. It is an important factor to consider and react accordingly to the students’ affective conditions, and traditional teaching in a classroom is a success there. In traditional classroom teaching, a teacher can change his/her strategy of teaching by observing the students’ facial expressions, emotions and body movements. Nevertheless, in e-Learning or online learning contexts, this becomes challenging.

In human society, the appearance of individual perceptions and responses is called emotions. It is an elemental part of the individual that affects their behavior, decision-making, ability to think, adaptability, well-being and the way they interact with each other [4,5]. Emotions have a significant impact on human actions in individual and social cultures and need to be examined in human practices, such as e-learning [6]. In [7] researchers investigated the influence of experimentally induced positive and negative emotions on multimedia learning (education context). Their result has shown that learners having the highest prior knowledge or working memory capacity have compensated the emotional impact on learning outcomes.

Recognizing and responding appropriately to the affective conditions of students is an essential aspect, and conventional classroom teaching is successful. Teachers may adjust their teaching strategy in traditional classroom learning by observing the behavior, facial expressions, and body gesture of the students. Nevertheless, this is quite difficult for the e-Learning context. According to [8,9], it is not just learning but the inter-dependency between learning and emotion that is mediated in the e-learning context of social learning (teachers, students and learning). From the learners’ point of view, the desired favorable outcomes are proper decision making, learning activities management and time management; combining all these outcomes will enhance students’ attention, motivation and desire to learn.

Human emotions are identifiable using behavior, vocal, facial expression or physiological signals. However, facial expression, behavior and speech are subjective [10]. For instance, a subject can purposefully mask their true emotion, which may be conflicting. In contrast, physiological signals are more reliable and unbiased in emotion recognition. One such very popularly used signal for human emotion recognition is electroencephalography (EEG). EEG signals are produced by the Central Nervous System (CNS) and respond more rapidly with respect to the emotion change. Besides, EEG signals have been used because they provide valuable features for emotion recognition.

### 1.1. *Research Problem*

Emotion recognition in e-learning is the domain of application where conventional Machine Learning (ML) approaches have primarily been used in offline mode, where data can be scanned unlimited times. These approaches have limitations in e-learning perspective, such as

- Emotion classification in e-learning needs real-time classification, but offline ML approaches are incapable of doing so.
- Offline ML emotion classifiers are data-hungry, and to perform complicated emotion recognition, appropriate training data are needed. Those training data are immutable, meaning it can be viewed indefinitely. Researchers use the historical data in current research to train the ML model (i.e., offline mode training) and then use trained models to evaluate emotions. In real-time emotion classification scenarios, such cases will be costly and infeasible.

The aim of this research study is to develop a real-time emotion classification system that can classify the emotional states using a physiological data stream. We propose a real-time emotion classification system (RECS), which is developed by using Logistic Regression (LR) trained in online fashion using the Stochastic Gradient Descent (SGD) algorithm.

Specifically, our motivation and aims are as follows:

- To circumscribe the study in the field of online classifiers for emotion classification from an EEG data set.
- To refer and compare to existing research works that used the DEAP data set, which is a commonly used data set in the field of emotion classification.
- To build a lightweight emotion classifier that could be very efficient so that emotions can be classified in real time and so could be used in real life contexts where time dimension is of importance, such as in e-Learning.
- To build a lightweight emotion classifier useful for practical applications in a realistic scenario of a client-server distributed system: the client is the EEG sensor, while the server is a smart phone, any edge/fog computing device “close” to the user, where data are generated.
- The achieved accuracy of our approach is deemed reasonable in our context of the augmented workspace project where our emotion classifier will be incorporated.

### 1.2. This Paper Makes the Following Contributions

1. We have developed a real-time emotion classification system that uses Logistic Regression (LR) trained in an online fashion using the Stochastic Gradient Descent (SGD) algorithm. In our case, we have used EEG data as the physiological data stream. The EEG data come in a stream, and the emotional state is classified in real-time.
2. We have demonstrated that our proposed RECS classifier can outperform state-of-the-art online streaming classifier approaches; namely, we have considered five online classifiers: Hoeffding Tree (HT), Adaptive Random Forest (ARF), Dynamic Weighted Ensemble (DWE), Additive Expert Ensemble (AEE) and Hoeffding Adaptive Tree (HAT). We have also compared eight offline mode machine learning approaches, including Support Vector Machine (SVM), Multi Layer Perceptron (MLP) and Decision Tree (DT), which are implemented by ourselves, and five online classifiers from the literature (Naive Bayes, SVM, Hidden Markov Model (HMM) and K-NN) from the literature. It is to be noted here that comparing with offline classifiers is not the primary objective of this work, and therefore, we have not considered computationally heavy learning approaches such as ones based on deep learning.
3. We have analyzed the proposed approach in the context of an application scenario (e-learning application) where real-time emotion classification from an EEG data stream can be incorporated.

The rest of the paper is organized as follows: in Section 2 and Section 3, related works and preliminaries of various concepts such as data stream and emotion representation are introduced, respectively. In Section 4, materials and methods related to our proposed approach are presented. Analysis of results and evaluations are presented in Section 5. An application scenario is presented in Section 6. Section 7 draws the conclusion. Finally, in Section 8 the limitations and future scope of our work are discussed.

## 2. Related Works

EEG streaming has become popular in the research field of emotion detection and classification in various contexts. In the following we refer to research works that are related to our work because they classified emotions for use in e-Learning and education contexts. Most of them used an EEG data set, although in some cases the EEG data set was used as part of a multi-modal approach, where other data such as facial expressions, movement and body gestures are used for emotion classification. In addition, different classification methods are employed such as Fuzzy-based, SVM, KNN or ANN.

In [11], EEG signals and facial expressions have been used to recognize emotions. Researchers used a method of 3D fuzzy feature engineering to excerpt characteristics from EEG signal and video. It is called a 3D Fuzzy GIST. To collect emotion features and low-level video details (color, orientation), researchers have used their proposed approach. The 3D fuzzy tensor has been utilized to retrieve emotion features from EEG for subjective awareness of emotions. For concatenating two modalities, the feature-fusion approach has been utilized. The Adaptive Neuro-Fuzzy Inference System has been utilized in their research to classify two emotional states, positive valence or negative valence, on the basis of the feedback emotions deduced by a film clip from the subject. The proposed system has been integrated into a cognitive architecture where a humanoid robot interacts with humans.

In [12], a multi-modal emotion recognition system that has used video (an external channel) for facial expression and EEG (as internal channel) signals. To reduce the dimensionality, researchers have used a sequential floating forward scan method, and the emotion classification has been performed using two machine Learning (ML) approaches called Support Vector Machine (SVM) and K-Nearest Neighbors (KNN). Finally, a fusion between external and internal networks has been performed to the level of function and decision to evaluate emotions in the arousal dimension for multi-modal emotion recognition.

In [13,14], the authors have shown that by integrating emotional states into the learning management system, learners’ understanding, engagement and motivation will improve. Additionally, emotional information opens up the possibility to extend the use of educational technology and to offer potential incentives for enhancing e-learning outcomes (such as new and easily understandable content preparation, customized and personalized learning). It should be stressed that the authors in [13,14] did not process an EEG data set, yet their experiment is useful for integrating the EEG-based emotion classifiers into an e-Learning management system.

In [15], researchers have focused on how students can be engaged in e-learning for a more extended period. The emotional state recognition offers adaptive content about a learner’s emotion status, subject knowledge, profile and feedback. The learners’ emotion recognition been performed using facial expressions, movement, body gestures and EEG signals. Their study on recognizing the emotional state has shown that the bio-signal is a reliable channel for predicting learners’ emotional states. The authors use Feed-Forward Neural Network with back propagation for their emotion classifier.

Recently in [16], we have introduced data stream processing approaches for emotion classification, and we also reviewed various emotional states, which are mostly studied for the e-Learning context. Furthermore, we have identified the most appropriate data channels for emotion recognition. A brief analysis of emotion classification using physiological data (EEG) stream using Massive Online Analysis (MOA) [17] is also presented.

Finally, we refer to some recent works [18,19] where new types of EEG sensors are presented. These works are interesting in our context because they present a new generation of EEG sensors, namely EEG head band. The use of EEG head band is much more friendly and acceptable by users (e-Learners in our context) than traditional EEG artifacts. Different versions of EEG head band exist, and some of the most popular are the Emotiv Insight, Muse InteraXon, Emotiv Epoc+ and Neurosky Mindwave (refer to Section 6 for some pictures). In [18], researchers have used the EEG for human activity recognition. A Deep Learning-based framework is proposed to classify EEG data, based on a person’s physiological activity. In their deep learning framework, a Convolutional Neural Network and a Long Short-Term Memory Recurrent Neural Network are used to classify raw EEG signals based on the EEG artifacts. In [19], authors have explored the utility of lightweight, affordable and easy-to-set-up EEG systems to use in school-based research with children. The effects of a deep-breathing-for-test-anxiety intervention on brain electrical activity in eleven-year students are examined. Their study is based on power spectral analyses.

### 2.1. Other Related Work

We have analyzed various other works in the literature for offline approaches based on deep learning, CNN, RNN, etc. These works are indeed very interesting and could outperform our online classifier, but these are deemed computationally heavy methods and are not suitable for lightweight online classifiers.

Among such works, there are the following ones.

The authors in [20] have proposed a six-layer biologically inspired feed-forward Neural Network. For evaluation, they do not consider the DEAP data set, which is generated ad hoc from the authors and is different from the standard DEAP data set. It should also be noted that the e-Learning context was not considered in their work. Actually, the output of the study is that the radial basis function provided the best performance in their Neural Network approach. Their approach is therefore not suitable for online classification as it is computationally costly due the complex layered model used.

In [21], emotional states are classified from EEG data using a Long Short-Term Memory (LSTM) network. The researchers have used the DEAP data set for their study. However, there are two aspects to point out here: the e-learning context was not considered, and they present a model based on LSTM, which is more complex than standard recurrent nets and, therefore, has increased complexity.

In [22], authors aimed to classify emotions automatically for brain-inspired robot interaction with humans. Their novel framework for emotion recognition using multi-channel EEG consists of a linear EEG mixing model and an emotion timing model. The Stacked Auto Encoder (SAE) is used for the linear EEG mixing model, and for the emotion timing model, a combined Long Short-Term Memory Recurrent Neural Network (LSTM-RNN) is used. Their proposed framework has achieved better accuracy for emotion classification from the DEAP data set. However, the considered model is complex, and heavy computation time is required to train and test those models, making the model unsuitable for online classification.

In [23], 3D-Convolution Neural Network (CNN) is used for emotion classification from EEG data. In their approach, they have developed a data augmentation phase to enhance the 3D-CNN model’s performance. It should be noted that the considered CNN model, along with the data augmentation phase, makes the model more complicated and of high time complexity. Therefore, their approach is unsuitable for online classification.

In [24], the authors have used a multi-column CNN model for emotion classification from EEG signals from the DEAP data set. Their best results are achieved after considering many columns (about 7) in the CNN architecture. The main noticeable point here is that CNN is a Deep Learning approach with many convolution layers and fully connected layers stacked into it. The proposed model is not suitable for online classification because its complex structure makes the computation more expensive.

## 3. Preliminaries

In this section, preliminaries of various concepts related to the data stream, learning from the data stream and most relevant emotional models are introduced.

### 3.1. Data Stream

A data stream (DS) is a potentially unbounded, ordered sequence of data items, which arrive continuously at a speed. The main characteristics of data streams, according to [25], include

- Continuous flow: tuples are arriving one after another;
- Volume: huge data volume (i.e., infinite length);
- Velocity: fast arrival means relatively high speed with respect to the processing speed;
- Variety: data distributions generating may change on the go (dynamic change).

The data stream can be formally defined as a sequence of data samples or observations: $DS=\left\{x_{1},\;x_{2},\ldots ,x_{t},\ldots \right\}$, where $x_{i}$ is the $i^{th}$ arrived observation of data or data object. In this work we consider the case of labeled data streams, where each data sample or observation $x_{i}$ has a class label $y_{i}\in Y=\left\{y_{1},\;y_{2},\ldots \ldots ,y_{c}\right\}$.

### 3.2. Learning from a Data Stream

**Figure 1:**
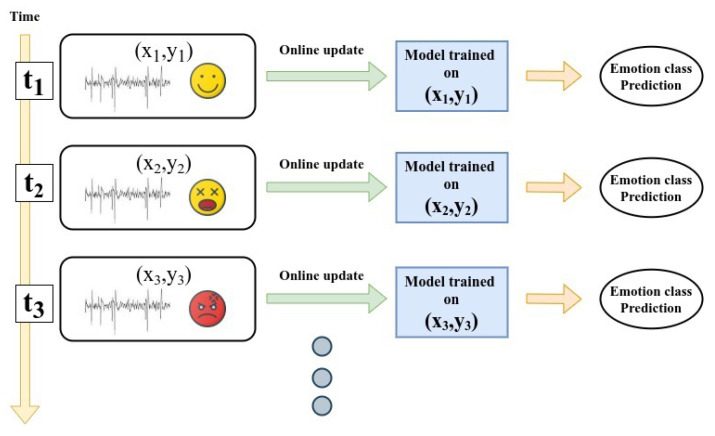
Online emotion classification from a data stream.

In stream learning, a model learns gradually from the tuples as soon as they arrive, and a single pass through them is performed [26] (see Figure 1). Here it is important to note the time dimension in Figure 1, where different tuples arrive in a stream mode at different points in time ($t_{1},\;t_{2},\ldots )$. Thus, there is one model that processes the stream. As soon as the data arrive, the model will be tested using the previous model. Then, the training will be performed based on the error, and a new model will be there for the next data; this process will go on as long as the data stream is arriving. In the beginning the model will perform very poor because the model has no knowledge, and gradually it will learn and update itself from the data stream and perform better in classification. In addition, the model can be evaluated at any point in time, and there will be no loop back over any portion of the data set.

### 3.3. Emotion Representation

Defining various emotions in a meaningful way is essential, and it has been a constant challenge for emotion researchers. The number of categories of emotion representation has always been controversial in psychology [10]. Researchers have focused widely on two emotion representation models deployed by psychologists: categorical or discrete emotion model (CEM) and dimensional emotion model (DEM) [27].

This dimensional emotion model (DEM) holds human emotion in dimension structure, where each dimension represents a characteristic of emotion. In multi-dimensional space, each emotion can be placed as a continuous and coordinated point in 3D or 2D. Instead of choosing discrete emotional state labels, a person’s impression can be indicated on a variety of continuous or discrete scales such as pleasant–unpleasant, attention–rejection or valence–arousal–dominance (VAD) [28]. The most widely used model under DEM is VAD, where valence is a satisfaction varying from positive to negative, arousal reflects the strength of feelings ranging from calm to excited, and dominance is the degree of control that varies from controlled to in control. Dominance is difficult to calculate and is mostly omitted, leading to the two-dimensional valence–arousal (VA) space commonly used [29]. Apart from these, there are many multi-dimensional emotional models present in literature. Typical examples are

- Russel’s early circle 2D model: arousal and valence are used for plotting emotion labels;
- Whissell’s continuous 2D space: dimensions are evaluation and activation;
- Schloberg’s 3D emotion model: attention and rejection dimensions are added to the 2D model.

**Figure 2:**
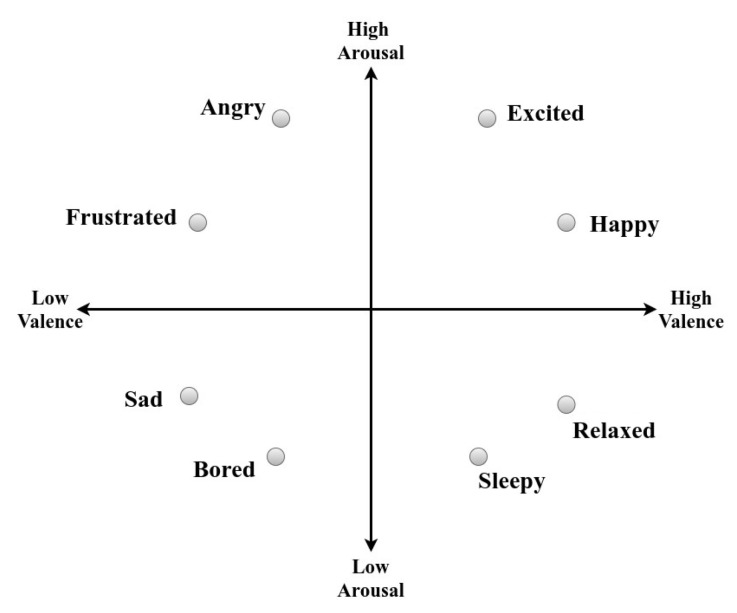
The Russel 2D valence–arousal (VA) space emotion model.

The commonly used model in DEM is Russell’s 2D emotion model. Figure 2 represents a schematic view of 2D model/VA space model.

## 4. Materials and Methods

As stated earlier, the objective of this works is to build an online classifier for the emotional states of learners and analyze its performance in comparison with other online and offline classifiers in the literature. To this end, we consider the Logistic Regression as a learning method trained in an online fashion using Stochastic Gradient Descent. The classifier uses the DEAP data set, which has become a standard data set in this field. The performance of the proposed online classifier is then compared to the state-of-the-art in the field.

Next, we present the DEAP data set [30], the working principles of LR, the stochastic gradient descent (SGD) optimizer and incremental SGD and feature extraction from EEG signals. Furthermore, we describe in detail the proposed methodology, the experimental setup and finally the performance metrics for model evaluation.

### 4.1. Data set Description

For emotion analysis and recognition, the DEAP (https://www.eecs.qmul.ac.uk/mmv/datasets/deap/ accessed on 19 February 2021) [30] (Database for Emotion Analysis using Physiological Signals) is a widely used, free, multimodal data set among emotion researchers. The data set contains EEG, peripheral and video signals. The data were recorded at two distinct locations Geneva (Switzerland) and Twente (Netherlands). The experiment was conducted on 32 participants in which 16 were male and 16 female. Participants 1–22 were recorded in Twente and participants 23–32 in Geneva. Each participant watched 40 different music videos of 60 s in length. In the experiment, a total of 48 channels including 32 EEG channels, 12 peripheral channels, 3 unused channels and 1 status channels were used to record the raw data. After finishing each video, each participant was asked to give the rating in terms of valance, arousal, dominance and familiarity ranges from 1 to 9 and liking ranges from 1 to 5. There are 32 files, one file for each participant’s recordings. Each trial contains 63 s signals, the first 3 s are the baseline signals. The data were down-sampled from 512 Hz to 128 Hz, and a bandpass frequency filter from 4.0–45.0 Hz was applied. Each data file is stored in a 3D matrix representation (40 × 40 × 8064), which represents video/trial × channel × data. The emotion labels are stored in a 2D matrix (40 × 4) in the same file.

### 4.2. Logistic Regression

Here we start by presenting the working principle of the Logistic Regression (LR) method. At the end of this subsection, we highlight the main reasons and the motivation for using LR as a basis for our online emotion classifier.

Logistic Regression (LR) is considered as a generalized linear model because the output always depends on the sum of the inputs and parameters. Let us consider a labeled data set *D*, where an *m* dimensional feature vector of *n* data points presents $D={\left\{\left(x_{i},y_{i}\right)|x_{i}\in {ℜ}^{n},y_{i}\in \left\{-1,1\right\}\right\}}_{i=1}^{m}$. $x_{i}$ is the *m* dimensional feature vector, and $y_{i}$ is the corresponding class labels. In logistic regression, instead of minimizing the linear objective function (e.g., sum of squared error (SSE)), a log-likelihood objective function is minimized. The sigmoid function, which outputs the conditional probabilities of the prediction (the class probabilities) is as follows:

(1) $$\sigma \left(z\right)=\frac{1}{1+e^{-z}}$$

where *z* is defined as the inputs and can be formulated as:

(2) $$\begin{array}{c} z=w_{0}x_{0}+w_{1}x_{1}+\ldots \ldots +w_{n}x_{n}\\ =\sum_{i=1}^{n}w_{i}x_{i}\\ =W^{T}X \end{array}$$

The sigmoid function takes *z* and then transforms it into the range of [0,1]. After model fitting, or in other words model training, by adjusting the weights by minimizing the cost or objective function [31], the step function converts the output from the activation function into binary class levels(*y*) as follows:

(3) $$y=\left\{\begin{array}{c} 1\;if\;\phi (z)\geq 0.5\\ 0\;else \end{array}\right.$$

An objective function is a single scalar value that is formulated from a set of design responses (https://abaqus-docs.mit.edu/2017/English/SIMACAEANLRefMap/simaanl-c-optobjectives.htm accessed on 19 February 2021). The log-likelihood objective function of logistic regression can be formulated as follows [32]:

(4) $$J\left(w\right)=\sum_{i=1}^{n}-y^{\left(i\right)}\log\left(\phi (z^{\left(i\right)})\right)-\left(i-y^{\left(i\right)}\right)\left(1-\phi (z^{\left(i\right)})\right)$$

(5) $$J\left(\phi (z),y;w\right)=\left\{\begin{array}{c} -\log(\phi (z))\;\;if\;y=1\\ -\log(1-\phi (z))\;\;if\;y=0 \end{array}\right.$$

Here we minimize the logistic objective function (Equations (4) and (5)) of LR so that we can develop an accurate model for classification with minimum error. Basically, the cost function measures how well our parameter w is doing on the data. So, it seems natural to minimize the cost function for minimal error across the data set to find w. In the streaming scenario, the LR model will be trained incrementally, i.e., the error will be minimized based on the arrived data stream, and the model’s performance will improve gradually.

For our Real-time Emotion Classification System development, we have considered LR as the base emotion classifier, which will be tested and trained in an online fashion. The main reasons for choosing LR for our emotion classifier in online learning are as follows:

- It requires the observations to be independent of each other. In the RECS scenario the participants are watching different videos, and the corresponding EEG recordings are independent from each other;
- It makes no assumption about the distributions of classes in feature space;
- It is efficient at classifying unknown records;
- It is very efficient in training and is a less complex model (less computation required); therefore, it is well suited for online learning;
- In the literature of emotion recognition from physiological data the LR model has been widely used.
- Finally, our RECS approach follows the development of other similar online learning approaches that were evolved from offline learning approaches. For instance, Adaptive Random Forest, Hoeffding Tree and Hoeffding Adaptive Tree are online versions based on offline methods of Random Forest and Decision Tree. Thus, here we are proposing RECS as an online classifier evolving from an LR offline classifier.

### 4.3. Stochastic Gradient Descent

In supervised learning, each data point in data set *D* represents a pair (x,y) consisting of input features *x* and scalar class labels *y*. An objective function $L(\hat{y},y)$ is considered to measure the difference between neural network predicted class labels (considered as $\hat{y}$) and the actual class labels *y*. A family F of functions $f_{w}\left(x\right)$ is parameterized by weight vector *w*. The function f∈F minimizes the loss or error $Q\left(D,w\right)=L(f_{w}\left(x\right),y)$ averaged on the training samples [33].

(6) $$E_{m}\left(f\right)=\frac{1}{m}\sum_{i=i}^{m}L\left(f\left(x_{i}\right),y_{i}\right)$$

The empirical risk $E_{m}\left(f\right)$ is to be minimized. It tests the efficiency of the model on the training set.

In practice, most often the empirical risk ($E_{m}\left(f\right)$) is minimized by the gradient descent (GD) approach. The weights *w* are updated based on the gradient of the empirical risk ($E_{m}\left(f\right)$). The weight change is formulated as follows:

(7) $$w_{t+1}=w_{t}-\eta \frac{1}{m}\sum_{i=1}^{m}\nabla_{w}Q\left(D_{i},w_{i}\right)$$

where η signifies the learning rate or step size. In practice, the learning rate value is set between 0 and 1. Researchers have noticed that a large learning rate (η) makes the convergence too fast, having less exploration, which implies bad generalization; on the other hand, small η makes the algorithm converge too slow, which means more exploration and implies good generalization but less exploitation. Therefore, in practice, researchers try to balance exploration and exploitation by setting the learning rate (η) properly. Mostly, it is selected with experiments, but the range should be between 0 and 1.

When the training data set is large the gradient descent algorithm is most likely to be ineffective. The ineffectiveness is rectified using the Stochastic Gradient Descent (SGD) algorithm. The stochastic version of the GD algorithm is SGD. In SGD, the gradient of $E_{m}\left(f\right)$ is not calculated exactly; instead, the gradient is estimated based on the randomly selected single data sample $D_{i}$ in each iteration:

(8) $$w_{t+1}=w_{t}-\eta_{t}\nabla_{w}Q\left(D_{i},w_{i}\right)$$

The stochastic process $\left\{w_{t},t=1,\ldots \ldots \right\}$ depends on randomly selected data samples in each iteration.

### 4.4. Incremental Stochastic Gradient Descent

In practice, to train a model properly in supervised learning one need to pass through the training data set multiple times (called epochs). The batch mode gradient descent approach may be infeasible and create an overhead in an environment where data are non-stationary, i.e., for streaming data. Because in streaming data the number of observations increases and data have velocity, batched mode gradient descent operations are expensive to perform in an online scenario [34].

(9) $$w_{i}=w_{i-1}-\gamma_{i}\nabla V\left(\langle w_{i-1},x_{t_{i}}\rangle ,y_{t_{i}}\right)$$

The main difference with the stochastic gradient method is that here a sequence $t_{i}$ is chosen to decide which training point is visited in the $i^{th}$ step. Such a sequence can be stochastic or deterministic.

To train the LR in an online fashion, we have used an incremental SGD trainer. In RECS we use EEG stream data where the classifier can be trained exactly once. Incremental SGD is therefore appropriate in this scenario because the LR model will be trained once, and it calculates the gradient on exactly one data sample to decrease the error so that the performance of the classifier will increase gradually.

### 4.5. EEG Feature Extraction

Feature extraction is crucial for extracting information from EEG, which reflects the emotional state very effectively. Then, those extracted features are used to train methods for emotion classification.

Wavelet decomposition (WD) is a standard, feasible and most effectively used time-frequency analysis method. It is the most popularly used feature extraction method applied on EEG signals because of its localized analysis approach that uses time as well as a frequency window, multi-rate filtering, multi-scale zooming, and is best suitable for non-stationary signals [35]. The multi-scale analysis of EEG signals provides both details and approximations of the EEG signal at different wavelet scales [10]. It also provides a series of wavelet coefficients at different scales. All these coefficients are capable of describing the original signal’s complete characteristics; that is why these are considered features of the signal.

Let us consider a given signal X(t); its WD can formulated as follows:

(10) $$X\left(t\right)=\sum_{k=-\infty }^{\infty }C_{n,k}\phi \left(\left(2^{-n}t\right)-k\right)+\sum_{j=1}^{n}\sum_{k=-\infty }^{\infty }D_{j,k}2^{-j/2}\psi \left(\left(2^{-j}t\right)-k\right)$$

where

- $C_{n,k}$ represents kth approximate component of nth level of wavelet decomposition;
- $D_{j,k}$ denotes the kth detail component of jth level of decomposition;
- ψ(t) is the wavelet function. In general it is denoted as ψ(·) and is formulated as follows:
  (11) $$\psi_{a,b}\left(t\right)=\frac{1}{\sqrt{a}}\psi \left(\frac{t-b}{a}\right)$$
  where *a* and *b* represent the scale factor and time shift, respectively;
- ϕ(t) represents the correction coefficient.

There is a huge variety of wavelet base functions, and those functions have distinct characteristics in terms of smoothness, symmetry, compact factor and orthogonality. Most commonly, wavelet base functions are Meyer wavelet, Morlet mother wavelet, Haar mother wavelet and Daubechies wavelet [36].

In the literature, the most significant features extracted from an EEG signal from each channel using the wavelet coefficients of each sub-band include wavelet energy (Equation (12)), wavelet energy ratio (Equation (13)) and wavelet entropy (Equation (14)), which are formulated as follows:

(12) $$E\left(i\right)=\sum_{j=1}^{n_{i}}D_{i,j}^{2}$$

(13) $$R\left(i\right)=\frac{E(i)}{\sum_{j=1}^{n}E(j)}$$

(14) $$W_{S}=\sum_{i=}^{n}R(i)\ln\left(R\left(i\right)\right)$$

where $D_{i,j}$ denotes the corresponding level’s wavelet coefficients shown in Equation (10).

### 4.6. Experimental Study

**Figure 3:**
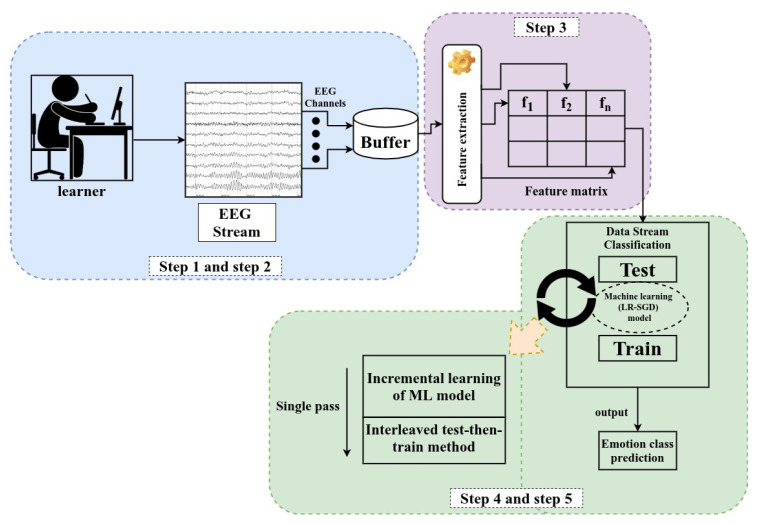
The framework of our proposed work.

In this section, the working procedure of real-time emotion classification using an EEG sensor data stream is presented. In addition, the schematic view of our proposed approach is shown in Figure 3. The experimental study is divided into the following steps:

Step 1: Data set consideration and data rearrangement: To make the EEG signal stream possible we have considered the pre-processed DEAP data [30]. As mentioned earlier, the DEAP data are stored in 3D matrix format, so for better understanding and readability we have rearranged the EEG signals into the one-dimensional (1D) matrix format as follows:
  $$\left[participant,video,channel\;no,channel\;data,valence\;class,arousal\;class\right]$$
  In this experiment, we have used the EEG data to classify emotions such as high/low valence and arousal. Thus, while streaming the valence and arousal scores present in the DEAP data set, an automatic scaling is done. For example, if valence is greater than or equal to 5, the class is high valence (i.e., 1); otherwise, the class is low valence (i.e., 0). A similar scaling is done for arousal class labels. In Table 1, the valence and arousal class distributions are shown.
  From Table 1 it can be seen that the data set is slightly imbalanced.
Step 2: Stream simulation: In the EEG stream simulation, we have considered a time window of 60 s for every participant. The reason for making the 60 s time window is that the videos are 60 s in length, and in DEAP data emotion labels are available for the corresponding video. We have considered that each participant is watching one video at a time, and the corresponding participant’s EEG signal stream is coming continuously. The data stream rate is 2 Mb/60 s. The streaming simulation system is developed using WebSockets, where from the client side continuous EEG data streams are coming to the server, and the server is processing the streams for real-time emotion classification.
Step 3: Feature extraction from the EEG signal stream: In our experiment, features are extracted from an EEG signal stream from each channel using the wavelet coefficients of each sub-band. The important features extracted are wavelet energy (Equation (12)), wavelet energy ratio (Equation (13)) and wavelet entropy (Equation (14)). In our experiment, we have used Daubechies wavelet and the near-optimal Wavelet Daubechies 4 (Db4) wavelet base as the wavelet base function to decompose the EEG signal into five levels.
Step 4: Emotion classification model: For emotion classification from the EEG stream we used a Logistic Regression model for high/low valence and arousal classification.
Step 5: Model test and training: The Logistic Regression model is trained in incremental learning using Stochastic Gradient Descent algorithm (Algorithm 1). The interleaved test-then-train [17] approach is used because this technique does not need separate memory for a test set and makes maximum use of the available data; the testing and training performance of the classifier can be examined with the most detailed possible resolution for each example. The meaning is that each individual example can be used to test the model before it is used for training, and from this the accuracy, FM score and confusion matrix are updated. The model is thus always being tested on examples it has not seen. In the initial phase (for the first data tuple) of interleaved test-then-train, the model will return nothing as it has no knowledge of the data, so the accuracy and *F1-score* will be zero. Then, gradually the accuracy and *F1-score* will increase by seeing more data tuples. The model is trained one time (i.e., the epoch is 1) for each EEG signal stream because, in streaming, the model can see the data once.

The following pseudo code is our proposed approach:
**Algorithm 1:** Pseudo code for the proposed real-time emotion classification using EEG stream
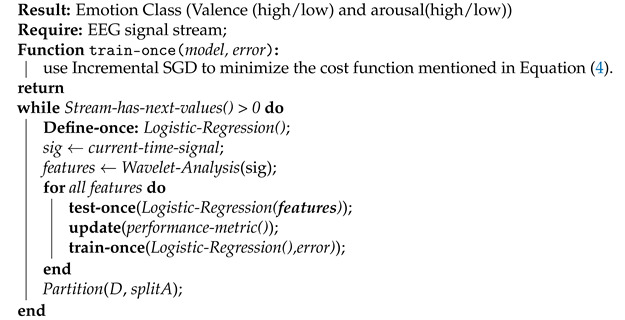


### 4.7. Experimental Setup

It is to be noted here that we have analyzed various other research works in the literature that build offline classifiers based on Deep Learning (see Section 2.1), CNN or RNN and that outperform our classifier, but these are computationally heavy methods, not suitable for lightweight online classifiers. Therefore, we have not included such offline classifiers in our comparison study.

Next, the machine setup, models used for comparison, model development and the parameter setup are presented.

**Figure 4:**
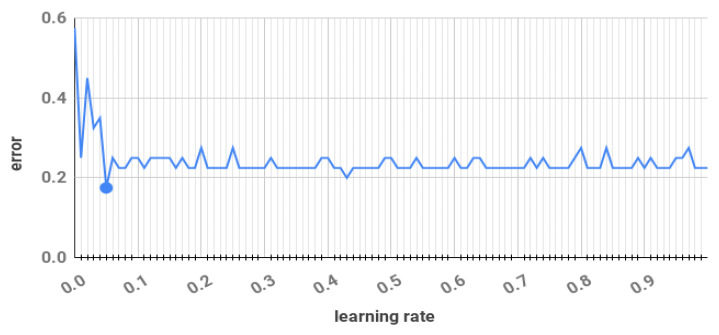
Learning rate vs model error.

1. Machine configuration: The machine configuration is Ubuntu 18.04 64 bit OS, processor core-i7-7700HQ with RAM 16 Gb–2400 MHz and 4Gb-Nvidia GTX-1050 graphics.
2. Compared models: To establish the performance of our model we have compared it with different popular offline mode ML-based emotion classifiers. We have considered Support Vector Machine (SVM) [37], Multi Layer Perceptron (MLP) [38] and Decision Tree (DT) [39] algorithms for the comparison. The reason for choosing the specified offline classifiers for our comparison is because these are the most popularly used emotion classifiers in the literature [10].
  To make the comparison more effective and clear, we have used five different streaming approaches for emotion classification using EEG data stream. All these approaches are effective for data stream classification approaches. The considered approaches are Hoeffding Tree (HT) [40], Adaptive Random Forest (ARF) [41], Dynamic Weighted Ensemble (DWE) [42], Additive Expert Ensemble (AEE) [43] and Hoeffding Adaptive Tree (HAT) [44]. The reason for choosing these approaches is that all these approaches are state-of-the-art online stream data classifiers. These approaches are the online version of Decision Tree and Random Forest (mostly used for offline mode emotion classifier). For instance, both HT and HAT are online versions of DT. Similarly, ARF is an online version of the Random Forest approach. We also did not come across any online emotion classifier that could be fairly compared to our approach. Therefore, we have considered these specified online approaches for emotion classification and compared them with our proposed model.
3. Algorithms development: The environment for RECS was developed from scratch using Python 3.7. Offline machine learning approaches (SVM, DT and MLP) were developed using Scikit-learn [45]. The online or streaming approaches (HT, DWE, ARF, AEE and HAT) were developed using a streaming Scikit-multiflow library [46].
4. Parameter setup: The considered machine learning approaches have various parameters that need to be configured. The parameters setup for offline classifiers (SVM, MLP and DT) were taken directly from emotion classification research papers [47,48,49]. The parameter setups are the following:
  - SVM: For SVM, the C (regularization parameter) is set to 1.0 and radial basis kernel (rbf) is used as specified in [49].
  - MLP: For MLP, one hidden layer with 20 neurons and sigmoid activation function is used. Stochastic Gradient Descent is used to optimize the MLP where the learning rate is 0.001. The maximum epoch is set to 200.
  - DT: For DT, everything is set to default as specified in [48].
  - RECS approach: In our approach, the learning rate for SGD is 0.05, and it is fixed throughout the emotion classification. To set the learning rate, we have done a *Cyclical Learning Rates* (CLR) [50] on one subject using RECS, where we have varied the learning rate for SGD from 0 to 1 with a step size increment of 0.01. The selection criterion for the learning rate of SGD is in which learning rate the RECS approach has less error (see Figure 4). From the plot, we can see that for the 0.05 learning rate of SGD, the RECS approach had 0.175 error, which is less in comparison to other errors. Note that the epoch is 1 because the model can see the data only once.
  - For all considered online approaches (HT, DWE, ARF, AEE and HAT) the parameters are set as specified in the corresponding papers [40,41,42,43,44].

### 4.8. Performance Metrics

For evaluating the classifiers performance in an imbalanced data set, even though our data set is slightly imbalanced, most popular performance metrics are F-measure (*F1-score*), balanced accuracy (*Acc*), confusion matrix and misclassification rate (mr) [51,52]. These metrics are calculated as follows:

(15) $$Acc=\frac{sensitivity+specificity}{2}$$

(16) $$F1-score=2\times (\frac{Pre\times Rec}{Pre+Rec})$$

(17) $$mr=\frac{\left(FP+FN\right)}{T_{s}}$$

where $sensitivity=\frac{TP}{TP+FN}$, $specificity=\frac{TN}{FP+TN}$, $Precision\left(Pre\right)=\frac{TP}{TP+FP}$, $Recall\left(Rec\right)=\frac{TP}{TP+FN}$ and $T_{s}$ is total number of samples.

- True positives (TP): actual class is positive and predicted class is positive.
- True negatives (TN): actual class is negative and predicted class is negative.
- False positives (FP): actual class is negative and predicted is positive.
- False negatives (FN): actual class is positive and predicted is negative.

F1-score is the weighted average of Precision (Pre) and Recall (Rre). Therefore, this score takes both false positives and false negatives into account. F1-score score ranges from 0 to 1: 0 means the worst classifier, and 1 interprets the best classifier.

A confusion matrix is a tabular way of visualizing the performance of the classifier. For binary classification, each entry in the principal diagonal of the confusion matrix denotes the number of predictions made by the model where it classified the classes correctly, and other entries denote that the classifier has classified the classes incorrectly.

For offline approaches, we have used 10-fold cross-validation, and for our proposed approach, we have tested 10 times to make the comparison very transparent. The average F1-score and Acc metrics are prioritized to establish the effectiveness of the proposed approach when it is compared with offline approaches. While comparing with streaming approaches, we have considered F1-score, Acc, confusion matrix and misclassification rate as our performance evaluation metrics.

## 5. Results and Discussion

In this section, we reported the comparison among traditional offline mode machine learning approaches, which are most frequently used for emotion classification, and we have utilized five different popular streaming approaches for valence arousal emotion classification from the DEAP data set.

It should be emphasized that we did not modify EEG data streams for using them in the offline algorithms. Indeed, while reading and processing the whole data stream already stored in the disk, we have stored the extracted features into the disk, and later, with the same extracted features, we trained and tested the offline approaches. Thus, the offline approaches see all data consisting of already all extracted features. On the other hand, for the online classifier, the feature extraction is done from the arrived data tuple from the stream, and then the classifier is tested and then trained (interleaved test-then-train) using the extracted feature. The whole process is repeated as long as the data stream is arriving. Therefore, in both offline and online approaches we are processing exactly the same stream, which makes the comparison reasonable.

### 5.1. Comparison with Online Streaming Approaches

**Figure 5:**
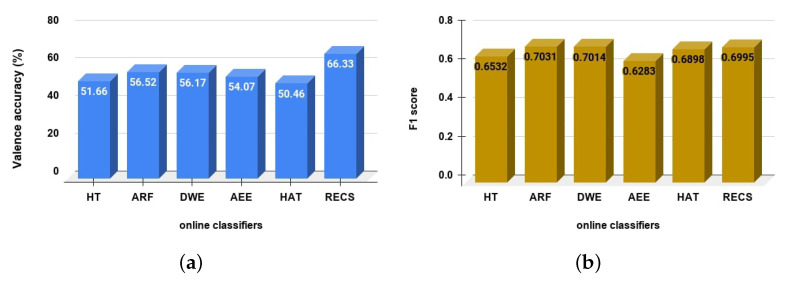
Valence classification comparison among different online classifiers. In (**a**) shows the accuracy and (**b**) shows the F1-score comparison.

**Figure 6:**
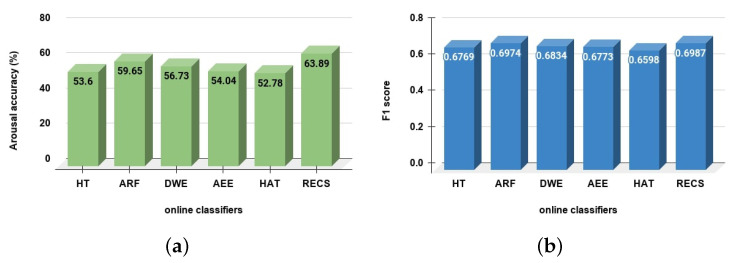
Arousal classification comparison among different online classifiers. In (**a**) is shown the accuracy and (**b**) shows the *F1-score* comparison.

In online or streaming approaches, the feature extraction approach (Wavelet decomposition) from the EEG data stream is the same for all considered online classifiers as well as our RECS approach. The classified emotions are valence (high/low) and arousal (high/low). In Figure 5 and Figure 6 the balanced accuracy and F1-score are presented for valence and the arousal emotion classification, respectively.

Figure 5a and Table 2 show the balanced accuracy for valence emotion classification. The RECS achieved 15.87%, 12.26%, 10.16%, 9.81% and 14.67% better accuracy than HAT, AEE, DWE, ARF and HT, respectively. Thus, RECS is has performed better in terms of valence emotion classification than other online classifiers. In addition, in the *F1-score* comparison (Figure 5b, Table 2), RECS outperformed HT, AEE and HAT classifiers in valence emotion classification. However, ARF and DWE achieved slightly better *F1-scores* of 0.35% and 0.18% than RECS.

From Figure 6a and Table 3, the balanced accuracy for arousal emotion classification the RECS achieved 10.9%, 4.24%, 7.16%, 9.85% and 11.11% better accuracy than HT, ARF, DWE, AEE and HAT, respectively. Thus, RECS is has performed better in terms of arousal emotion classification than other online classifiers. In addition, from the *F1-score* comparison (Figure 6b, Table 3) for arousal emotion classification RECS outperformed other online classifiers.

**Figure 7:**
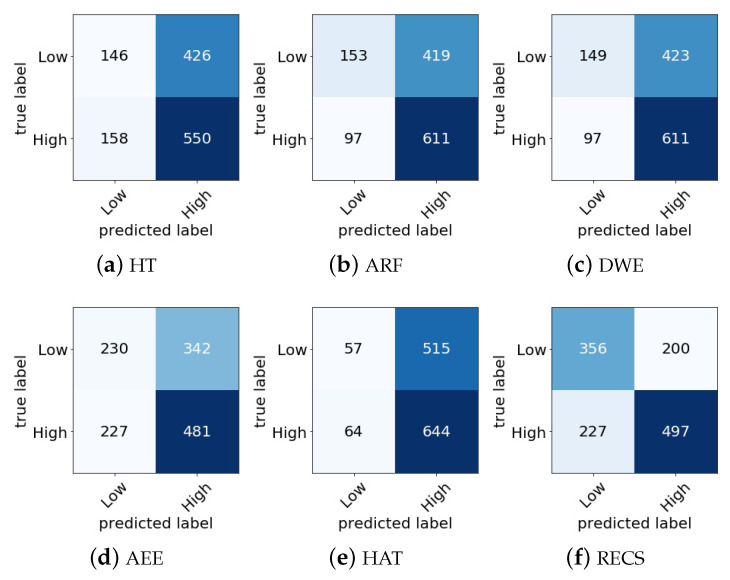
Confusion matrix comparison among different online classifiers for valence classification with our proposed RECS.

**Figure 8:**
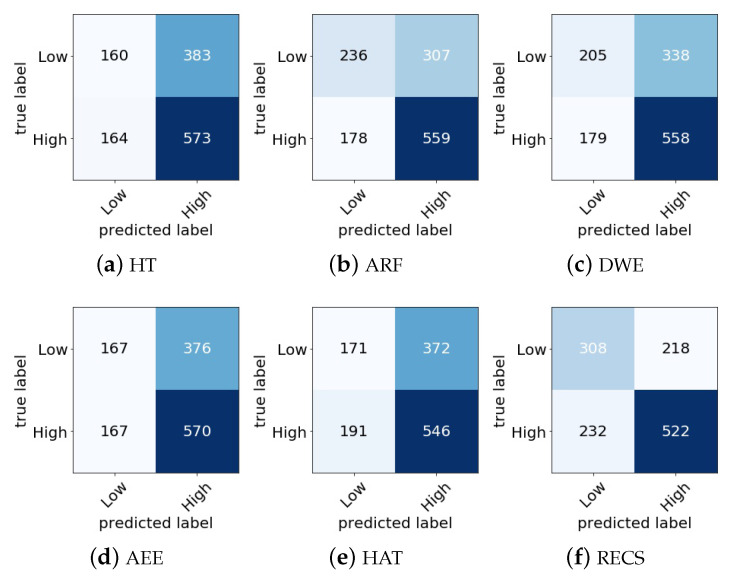
Confusion matrix comparison among different online classifiers for arousal classification with our proposed RECS.

In Figure 7 and Figure 8, the confusion matrix of different online classifiers as well as the RECS for valence and arousal emotion classification are presented, respectively. The principal diagonal elements in the confusion matrix show the correctly classified emotion classes, and other elements show the misclassified emotion classes. In the DEAP data set, the total number of valence classes was 1280, and for arousal 1280 classes. By referring Figure 7 and Figure 8, the misclassification rate in valence and arousal emotion classification was calculated for all online classifiers. The misclassification rates are shown in Table 4.

From the misclassification rate, Table 4, it can be seen that the RECS had lower misclassification rates of 12.27%, 6.96%, 7.27%, 11.1% and 11.68% than HT, ARF, DWE, AEE and HAT, respectively, for valence classification. For arousal classification RECS also showed lower misclassification rates of 7.58%, 2.74%, 5.24%, 7.27% and 8.83% than HT, ARF, DWE, AEE and HAT, respectively. Thus, from the comparison, it can be concluded that RECS performed better in terms of real-time valence and arousal emotion classification from EEG data stream because the misclassification rate of RECS was lesser than those of other online approaches considered for comparison.

### 5.2. Comparison with Offline Emotion Classifiers

We considered a total of eight offline classifiers for the purpose of the comparison study. These are organized into two groups: three offline classifiers (SVM, MLP and DT) implemented by ourselves and five online classifiers from the literature (Naive Bayes, SVM, Hidden Markov Model (HMM) and K-NN.)

#### 5.2.1. Comparison with Our Own Implemented Offline Classifiers: SVM, MLP and DT

The traditional machine learning approaches are trained and tested in offline mode, where the EEG signal is available for infinite time, with no memory restriction, no processing time restrictions, and multiple passes. Our proposed emotion recognition approach RECS is trained in an online fashion, where the EEG signal is coming as a stream, and the model can be trained using the data stream exactly once. The feature extraction approach (Wavelet decomposition) is the same for traditional ML approaches as well as our RECS.

We have considered for the sake of comparison three offline classifiers, namely, SVM, MLP and DT, which are commonly used for building online emotion classifiers. In this case, these methods are implemented by ourselves using Python libraries.

In Table 5 and Table 6, a comparison among all considered offline emotion classifiers with our proposed RECS in terms of average accuracy and *F1-score* is reported, respectively. In those tables, average (mean), maximum (max), minimum (min) and standard deviation (Std) are reported to show the models’ robustness. Lower Std indicates the model’s consistency in classification. The best accuracy and *F1-score* values are highlighted in bold.

From the accuracy comparison in Table 5, our proposed RECS achieved 15.13%, 12.48% and 13.14% better average accuracy than SVM, MLP and DT, respectively, for valence classification. For arousal classification RECS achieved 13.58%, 14.94% and 11.09% better average accuracy than SVM, MLP and DT, respectively. Another point to note is that for valence and arousal classification the Std was lower in our RECS than other offline classifiers, and with this we can conclude that our RECS is a robust model. Thus, in terms of average accuracy comparison our RECS approach has outperformed those offline emotion classifiers.

In Table 6, for valence classification, our proposed RECS achieved a better mean *F1-score* than DT and a similar mean *F1-score* with MLP, but in comparison with SVM, RECS achieved a slightly lower mean *F1-score*. In arousal classification, RECS outperformed all the offline emotion classifiers. In terms of model robustness, our RECS is much more robust than other offline approaches. Based on mean and Std of accuracy and *F1-score*, it is evident that RECS outperformed all the offline approaches for arousal emotion classification using EEG signal, and RECS is much more robust for emotion classification.

One reason that RECS has performed better than the compared methods is due to the imbalance data set. In the DEAP data set, the valence and arousal class labels are imbalanced (shown in Table 1). In [53,54] researchers have studied the effect of an imbalanced data set (i.e., class imbalance) on offline ML models. They have concluded that the class imbalance degrades the performance of ML/DL approaches. The same problem has happened in the offline classifiers, which are used for comparison. However, for the streaming cases, data are coming one by one, so there is no such case of data imbalance.

It is to be pointed out that in traditional SVM the class imbalance problem exists, and it impacts the classifier’s performance. However, the class-weighted SVM tackles the imbalance set issue. In our case, we have used traditional SVM with no class imbalance treatment. Similarly, for MLP class imbalance does affect the performance [55].

#### 5.2.2. Comparison with Offline Classifiers from the Literature

Our proposed RECS is compared with recent emotion recognition models presented in the literature [30,56,57,58,59], namely Naive Bayes, SVM, Hidden Markov Model (HMM) and K-NN. The state-of-the-art research articles are selected based on following criteria:

- In all these articles DEAP data set is used for emotion classification. The emotions are recognized from EEG data.
- Emotion classification of two classes (high/low valence and arousal) are done.

In [30], a Naive Bayes classifier is used to classify high/low valence and high/low arousal emotion classes from EEG data. Support Vector Machine classifier is used to classify high/low valence and arousal in [56,57]. In [59] a Hidden Markov Model is used to classify the valence and arousal emotions. An unsupervised approach called K-Nearest neighbor is used to classify the valence and arousal emotion class labels present in [58].

The comparison is shown in Table 7. The best results are boldfaced in the table.

From the comparison presented in Table 7, we can see that RECS has outperformed the state-of-the-art methods in the literature.

#### 5.2.3. Considerations for Other Deep Learning Based Approaches

There are other offline classifiers for emotion classification (please see Section 2.1) based on Deep Learning reported in literature. It should be mentioned these classifiers tend to be the most efficient ones, when compared to other more traditional classifiers, yet they are more complex and computationally expensive.

For Deep Learning approaches, as can be seen from Table 8 ([24]) Model for Emotion Recognition from EEG Signals. Sensors 2019, 19, 4736.), accuracy values of their Deep NN offline classifier are around 80%, on average, which outperforms our online classifier. However, for non-deep learning methods based on handcrafted features, their valence is at 71% on average, and arousal is 69% on average, which are similar to our online RECS performance values. These results confirm the challenge of achieving high accuracy values, and our results are close to those values.

## 6. Application Scenario

**Figure 9:**
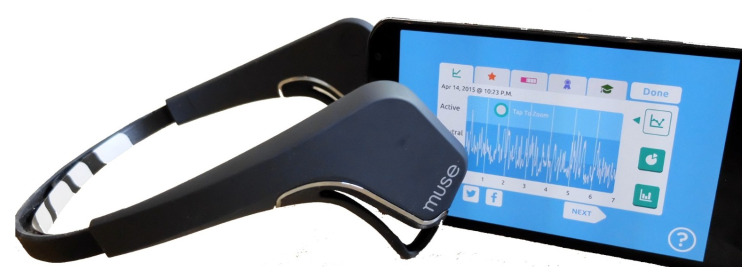
New generation of EEG head bands.

At present, the cost of EEG devices is significantly less than traditional EEG helmets, and their design is more user friendly [18,19]. A new generation of EEG sensors, namely EEG head bands, currently exist, such as Emotiv Insight, Muse InteraXon, Emotiv Epoc+ and Neurosky Mindwave (see Figure 9 for a muse EEG head band).

**Figure 10:**
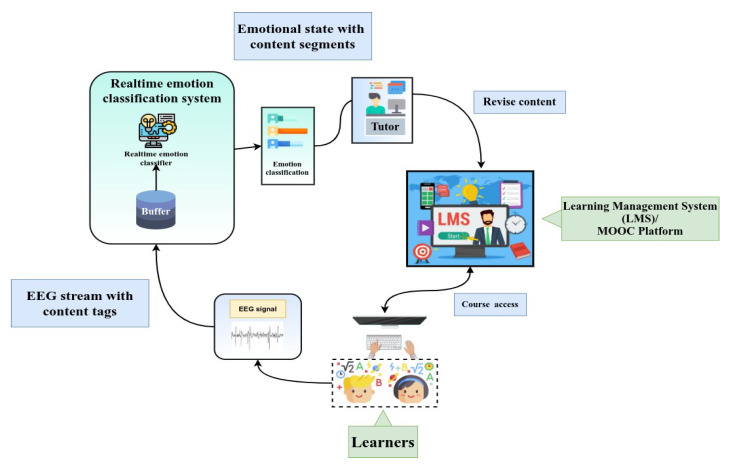
An application scenario.

Schools, colleges, individual users and online learning systems can afford to use EEG head bands for real-time emotional classification in different contexts. The EEG recordings can be transmitted via Bluetooth connected to his/her computer or smartphone. When a learner is watching a course in the e-learning portal or Learning Management System (LMS), she/he can wear the EEG device connected to the learning platform via Bluetooth. Then, the learner’s EEG data can be transmitted to the e-learning platform (see Figure 10).

Incorporating our developed real-time emotion recognition classification system can process the obtained data and then classify the learner’s emotional state needs to map arousal and valence to emotional states. After that, we can convert those valence and arousal labels into discrete emotion labels with the help of 2D VA space emotion model (see Figure 2 earlier in the text) as follows:

- Excited (high valence and high arousal)
- Angry (low valence and high arousal)
- Sad (low valence and low arousal)
- Relaxed (high valence and low arousal)
- Happy (high valence)
- Bored (low valence)
- Frustrated (high arousal)
- Sleepy (low arousal)

**Figure 11:**
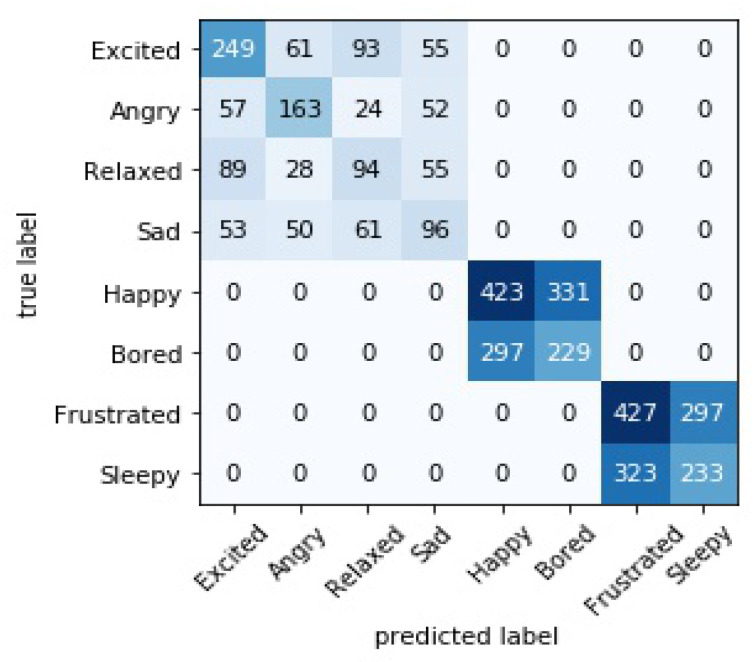
Confusion matrix for discrete emotion mapping based on valence–arousal status.

We have done a test on the discrete emotion mapping based on the valence-arousal status. The results in terms of accuracy, *F1-score* and confusion matrix are shown in Table 9 and Figure 11, respectively.

The system can label the content segment with the emotional states of a learner. By doing this, the tutor can easily identify in real-time the students having negative emotions (sad or angry), and a quick action can be taken to shift the learner’s negative emotion to positive (happy or relaxed) by counseling, giving more information, more details on learning exercise, scaffolding, etc. Identifying negative emotion at an earlier stage is a very crucial task. Emotion researchers in the learning domain aim to identify those learners having negative emotions while learning; otherwise, there is a high chance that those students could drop out of the course, which would affect their academic results. By understanding the emotional state’s classification in real-time using EEG data streams, tutors and students can improve and extend the use of educational technology and offer potential incentives for enhancing learning outcomes. This scenario can potentially be useful for learners as well as tutors in the e-learning context.

An application scenario of interest in this context is that of augmented workspace within Eurecat’s materials laboratory (Eurecat Technology Centre of Catalonia, Spain: https://eurecat.org/en/ accessed on 19 February 2021) where students perform learning tasks such as characterization of materials, erosion of the material, measuring the flexibility of materials, etc. The teacher shows the students the steps they have to follow for completing the learning exercise. According to the real-time emotion classification by RECS, the teacher will see the students’ emotional state in a dashboard. Then he/she will adapt the pace of the explanation—slowing down or accelerating—and pay attention to particular cases. Thus, the teacher will provide personalized support to those students who are not following as expected, and they will provide extra tasks to advanced students, thanks to knowing the emotional state of learners.

## 7. Conclusions

The effects of emotions on optimizing and enhancing e-learning outcomes have been studied by several researchers in the field. To accomplish this objective, several machine learning and deep learning methods have been proposed in the literature. However, the existing methods in the literature are data-hungry, and to extract emotion, more computations are required. Such methods are suitable for an offline mode where emotion recognition data are stored and can be retrieved infinitely, but these methods are inappropriate for e-learner’s real-time emotion monitoring. In this research, we have addressed this challenge by developing an online emotion classifier utilizing Logistic Regression (LR) trained in incremental fashion using the Stochastic Gradient Descent (SGD) algorithm for real-time emotion classification using EEG data stream. Based on the comparison results with offline emotion classifiers (such as SVM, MLP and DT) as well as streaming approaches (HT, HAT, ARF, DWE and AEE), we can conclude that our proposed approach for real-time emotion classification from EEG signal stream is effective in terms of significant average accuracy and *F1-score* improvement.

In summary, we have developed a real-time emotion classification system from the EEG data stream, which can classify emotion in real-time and outperform other offline and stream classifying approaches. We anticipate that our developed real-time emotion classification system can effectively classify the emotions of learners in real-time when they are learning from LMS and engaging themselves in the e-learning context.

## 8. Limitations and Future Works

In this work, we have used the DEAP data set. We would like to apply our approach to other data sets for e-learners’ emotion classification. Alternative data sets would give us more insights on emotion classification as well as for the suitability of using the emotion classifiers in e-learning contexts.

We also would like to study in more detail the concept drift change [60] happening in a data stream and how the model can be updated with the change so that the classifier’s performance can be improved. We will design a drift detector that can be combined with our classification algorithm to boost its accuracy beyond the current state.

In the future, we are also planning to conduct a study to collect data from learners in different contexts, and we will observe the performance of our proposed framework on the collected data set. We will also extend the emotional class study to a more granular number of classes to capture a more fine-grained emotion classification.

## Figures and Tables

**Table 1:** Valence and arousal class distributions in the DEAP data set.

Emotion Class Labels	Number of Instances in High	Number of Instances in Low
Valence	808	472
Arousal	816	464

**Table 2:** Accuracy and F1-score comparison among different online classifiers for valence classification.

Online Approaches	Performance Measures	
	Balanced Accuracy	*F1-Score*
HT	51.66	0.6532
ARF	56.52	0.7031
DWE	56.17	0.7014
AEE	54.07	0.6283
HAT	50.46	0.6898
RECS	66.33	0.6995

**Table 3:** Accuracy and *F1-score* comparison among different online classifiers for arousal classification.

Online Approaches	Performance Measures	
	Balanced Accuracy	*F1-Score*
HT	53.6	0.6769
ARF	59.6	0.6974
DWE	56.73	0.6834
AEE	54.04	0.6773
HAT	52.78	0.6598
RECS	63.89	0.6987

**Table 4:** Misclassification rate comparison among online classifiers.

Online Classifiers	Misclassification Rate for Valence	Misclassification Rate for Arousal
HT	0.4562	0.4273
ARF	0.4031	0.3789
DWE	0.4062	0.4039
AEE	0.4445	0.4242
HAT	0.4523	0.4398
RECS	0.3335	0.3515

**Table 5:** Accuracy comparison between offline emotion classifiers with our proposed RECS.

Emotion Classifier	Valence				Arousal			
	Mean	Max	Min	Std	Mean	Max	Min	Std
SVM (offline)	52.83	55.10	49.98	0.02	51.25	55.70	45.62	0.03
MLP (offline)	55.48	62.79	50.66	0.03	49.89	55.41	45.59	0.02
DT (offline)	54.82	60.49	49.17	0.03	53.74	58.52	49.57	0.02
RECS (online)	67.96	68.96	67.49	0.004	64.83	65.20	64.35	0.002

**Table 6:** *F1-score* comparison between offline emotion classifiers with our proposed RECS.

Emotion Classifier	Valence				Arousal			
	Mean	Max	Min	Std	Mean	Max	Min	Std
SVM (offline)	0.72	0.76	0.67	0.03	0.69	0.74	0.63	0.02
MLP (offline)	0.71	0.77	0.65	0.03	0.65	0.69	0.62	0.02
DT (offline)	0.60	0.67	0.54	0.04	0.58	0.64	0.54	0.03
RECS (online)	0.71	0.72	0.71	0.003	0.71	0.71	0.70	0.002

**Table 7:** Emotion recognition accuracy (%) comparison with recent models present in the literature.

Previous Research	Valence		Arousal	
	Accuracy	*F1-Score*	Accuracy	*F1-Score*
Naive Bayes [30]	57.60	0.563	62.00	0.583
SVM [56]	62.00	0.6845	62.67	0.7097
SVM [57]	64.90	0.5140	65.00	0.508
K-NN [58]	58.05	-	64.56	-
HMM [59]	58.75	-	55.0	-
RECS	67.96	0.71	64.83	0.71

**Table 8:** Performance accuracy results from Yang et al. [24] for Deep Learning vs. non-Deep Learning approaches.

Category		Valence	Arousal
handcrafted features	highest	76.9%	73.06%
	lowest	64.3%	66.20%
	difference	12.6%	6.86%
	average	71.66%	69.37%
deep neural network	highest	87.44%	88.49%
	lowest	72.06%	75.12%
	difference	15.38%	13.37%
	average	81.4%	80.5%

**Table 9:** Performance of RECS on discrete emotional state mapping using valence–arousal emotion status.

Emotions	Performance Metrics	
	Acc.	*F1-Score*
Excited	0.54	0.55
Angry	0.55	0.55
Relaxed	0.35	0.35
Sad	0.37	0.37
Happy	0.56	0.57
Bored	0.44	0.42
Frustrated	0.59	0.58
Sleepy	0.42	0.43

## Abbreviations

The following abbreviations are used in this manuscript:

Acc	Accuracy
AEE	Addditive Expert Ensemble
ANN	Artificial Neural Network
ARF	Adaptive Random Forest
CEM	Categorical Emotion Model
CNS	Central Nervous System
Db4	Wavelet Daubechies 4
DEAP	Database for Emotion Analysis using Physiological Signals
DL	Deep Learning
DS	Data stream
DT	Decision Tree
DEM	Dimensional Emotion Model
DWE	Dynamic Weighted Ensemble
EEG	electroencephalogram
FN	False Negative
FP	False Positive
GD	Gradient descent
HT	Hoeffding Tree
HAT	Hoeffding Adaptive Tree
KNN	K-Nearest Neighbors
LMS	Learning Management Systems
LR	Logistic Regression
ML	Machine learning
MLP	Multi-layer Perceptron
MOA	Massive Online Analysis
MOOC	Massive Online Open Courses
mr	Misclassification Rate
Pre	Precision
Rec	Recall
RECS	Real-time Emotion Classification System
SGD	Stochastic gradient descent
SSE	Sum of squared error
STD	Standard deviation
SVM	Support Vector Machine
TP	True Positive
TN	True Negative
VA	Valence-arousal
VAD	Valence-arousal-dominance
WT	Wavelet Transformation
